# A rare case of an HIV-seronegative AIDS patient with *Pneumocystis jirovecii* pneumonia

**DOI:** 10.1186/s12879-019-4143-8

**Published:** 2019-06-14

**Authors:** Shuangquan Yan, Jing Huang, Qiaofei Zheng, Hongguo Zhu, Zhuolin Gao, Jiaxi Feng, Youzu Xu

**Affiliations:** 1 Division of Respiratory Medicine, Taizhou Enze Medical Center Enze Hospital, No. 1 East TongYang Load, Luqiao District, Taizhou, Zhejiang 318000 People’s Republic of China; 2 Division of Clinic Laboratory, Taizhou Enze Medical Center TaiZhou Hospital, Taizhou, 318000 Zhejiang China; 3 Division of Clinic Laboratory, Taizhou Enze Medical Center Enze Hospital, Taizhou, 318000 Zhejiang China

**Keywords:** HIV-seronegative, AIDS patients, *Pneumocystis jirovecii* pneumonia

## Abstract

**Background:**

As technology progresses, several highly sensitive human immunodeficiency virus (HIV) screening kits are being researched and developed to quickly and efficiently identify serum HIV antibodies within the non-window period. In individuals who are HIV-seronegative, HIV infections that are not within a window period are rare. In such cases, all antibody detection methods will fail, and misdiagnosing these patients will have catastrophic consequences.

**Case presentation:**

A 22-year-old male Chinese patient with diffuse exudative lesions in both lungs and initial symptoms of cough and dyspnoea was diagnosed with *Pneumocystis jirovecii* pneumonia (PJP) by aetiological examination, and the patient’s plasma CD4^+^ T-cell count was extremely low. In China, PJP is prevalent in HIV-infected individuals. *Pneumocystis jirovecii (P. jirovecii)* has a high colonisation rate in patients with HIV infections. This patient was naturally suspected of being an HIV patient; however, serum HIV antibody tests were negative using both an enzyme-linked immunosorbent assay (ELISA) and a latex agglutination assay, and HIV was not detected by western blotting. Subsequently, the plasma HIV viral load was found to be extremely high on two repeated plasma HIV RNA tests, thus confirming HIV-seronegative acquired immunodeficiency syndrome (AIDS) in this patient. With administration of effective anti-*P. jirovecii* treatment and highly active antiretroviral therapy (HAART) after diagnosis, the patient’s disease condition was rapidly controlled.

**Conclusion:**

This is the second reported case in China of an HIV-seronegative AIDS patient. Such cases are also rare worldwide. Although HIV-seronegative HIV infections are rare, AIDS should be considered in immunodeficient patients with opportunistic infections, even if the test results are HIV-seronegative. Plasma HIV RNA testing is important for such patients.

**Electronic supplementary material:**

The online version of this article (10.1186/s12879-019-4143-8) contains supplementary material, which is available to authorized users.

## Background

*Pneumocystis jirovecii* (*P. jirovecii*) is an opportunistic pathogen with a high colonisation rate in human immunodeficiency virus (HIV) infections. *Pneumocystis jirovecii* pneumonia (PJP) is a severe lung disease caused by the massive proliferation of *P. jirovecii* in the lungs, and it is most common in acquired immunodeficiency syndrome (AIDS) patients. Identification of AIDS patients is valuable for diagnosing PJP. As technology has progressed, more methods of detecting HIV antibodies have emerged. Various detection methods have been developed and have become more sensitive. In China, the initial screening and diagnostic tests for HIV are mainly performed by applying different methods to detect serum HIV antibodies [1]. However, for AIDS patients who are HIV-seronegative, regardless of how advanced the serum antibody detection technology is, diagnosis will fail. Misdiagnosis or missed diagnosis of HIV infections will not only result in a lack of timely treatment for the HIV infection but will also confound diagnosing HIV infection-related diseases such as PJP, primary pulmonary Kaposi sarcoma (KS), and toxoplasma encephalopathy.

However, to date, HIV-seronegative AIDS cases are rare; only 27 cases have been reported globally. A recent case reported by Zhang et al. [2] was the first HIV-seronegative AIDS case reported in China. However, at least in China, the current practice is that nucleic acid testing is not required in HIV initial screening tests according to the guidelines [1]. With the increase in such reported cases, more importance will be attached to nucleic acid tests to screen out HIV infection.

Herein, we report an HIV-seronegative AIDS case that may lead to new arguments for HIV nucleic acid testing.

## Case presentation

**Fig. 1:**
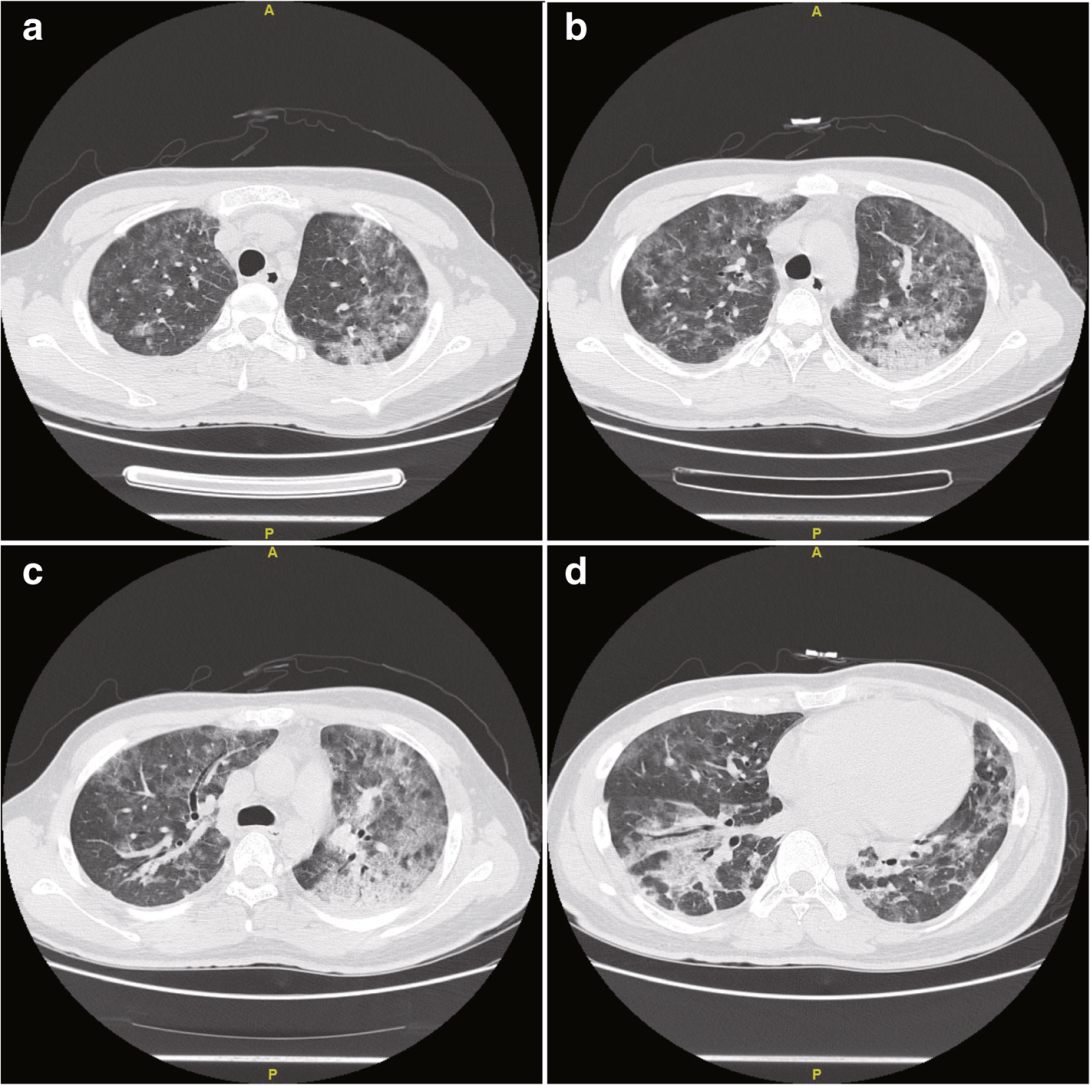
The patient’s first chest CT examination after onset, mainly showing filling and exudation of the diffuse alveolar cavity in both lungs (panels **a** through **d**), with thickening in some lobular septa

**Fig. 2:**
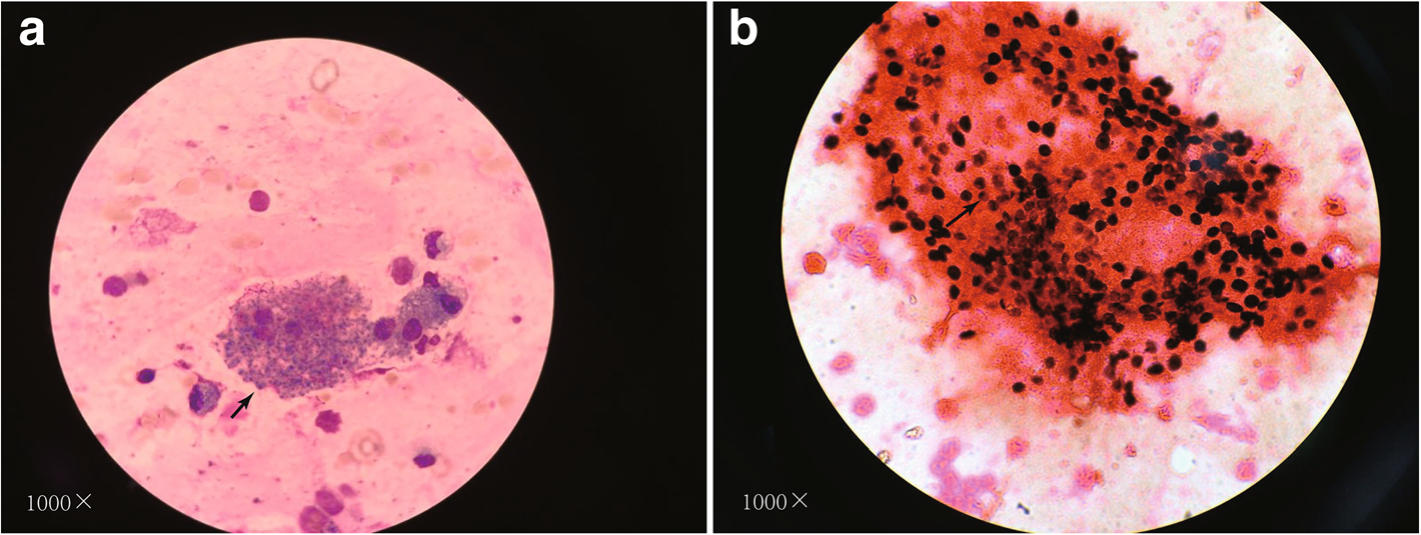
**a**: Wright’s stain, **b**: hexamine silver stain; the arrow indicates a *Pneumocystis jirovecii* cyst

**Fig. 3:**
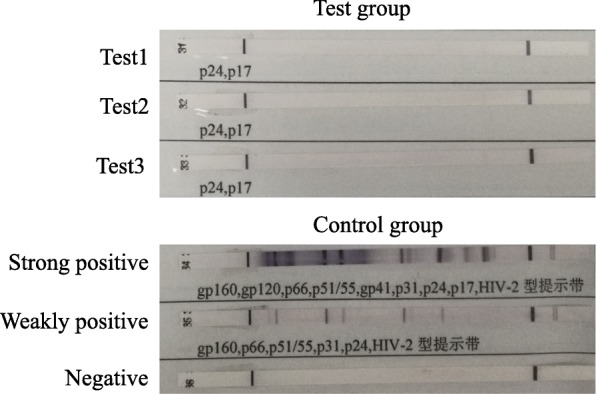
HIV antibodies in the plasma were detected by western blotting, but HIV antibodies could not be determined by three repeated experiments

**Fig. 4:**
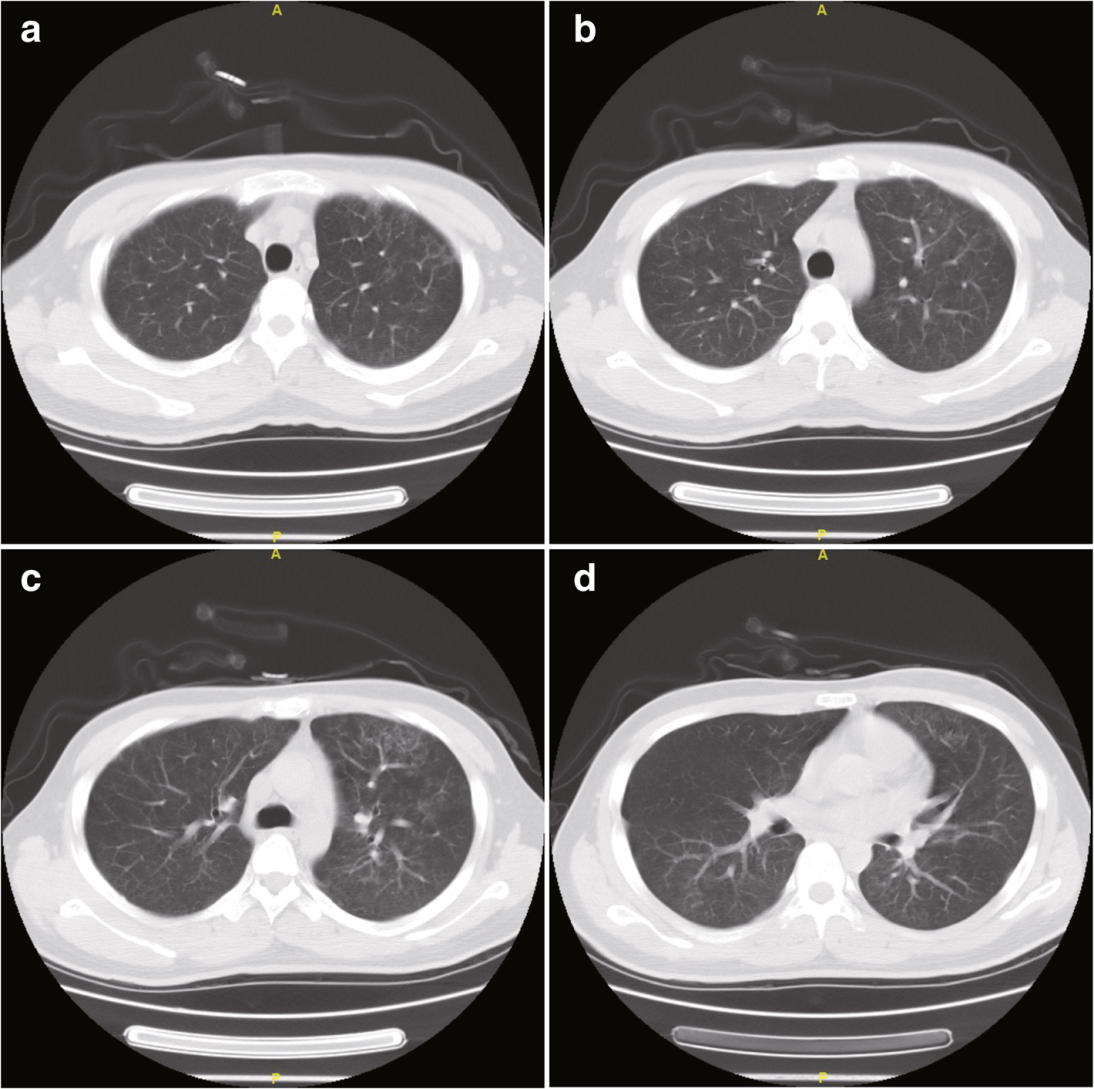
A CT scan of the chest was obtained after 12 days of anti-P. jirovecii treatment with SMZ-TMP and clindamycin, the diffuse lesions in both lungs were mostly absorbed (panels **a** through **d**)

On December 11, 2017, a 22-year-old Chinese unmarried male patient reported a history of male-male oral sex during high school. In June 2017, he underwent a peri-anal abscess operation at another hospital. No other history of anal sex, surgery, blood transfusion, dust exposure, or recent bird or poultry exposure was reported. Repeated coughing began more than a month prior, with a small amount of white sputum. He simultaneously began to experience shortness of breath after light activities, which was gradually aggravated. Two days before admission, the patient had a low fever, followed by no fever with cold and chills, and significantly aggravated dyspnoea, and he could not tolerate fast walking. An examination upon admission showed a white blood cell count of 6.9 × 10^9^/l, a neutrophil ratio of 83.8%, a lymphocyte ratio of 10.2%, a serum lactic dehydrogenase (LDH) level of 363 u/l, and a high-sensitivity C-reactive protein level of 77.00 mg/l. Arterial blood gas analysis showed a partial pressure of oxygen (PO_2_) of 62 mmHg, pressure of carbon dioxide (PCO_2_) of 33 mmHg and a pH of 7.43 without oxygen. An enzyme-linked immunosorbent assay (ELISA) was negative for HIV, and the results of other tests showed negative results for cytomegalovirus IgM antibody, positive results for cytomegalovirus IgG antibody, a CD4^+^ T-cell absolute value of 7.70/μl, a CD8^+^ T-cell value of 296.29/μl, a (1–3)-β-D-glucan level of 283 pg/ml, negative galactomannan and cryptococcal latex agglutination tests, an IgG level of 16.10 g/l, an IgM level of 1.36 g/l, an IgA level of 4.02 g/l, and an IgE level of 192.0 IU/ml. White *Candida albicans* growth was observed twice on sputum smear microscopy, and the sputum culture showed *C. albicans* growth once. Chest computed tomography (CT) showed diffuse ground glass-like lesions in both lungs (Fig. 1). The patient was initially treated with piperacillin tazobactam sodium injection + azithromycin injection + fluconazole injection + methylprednisolone injection, and his cough and shortness of breath improved slightly. PJP was suspected, and alveolar lavage was performed. The cellular proportions in the bronchoalveolar lavage fluid (BALF) were 62% phagocytic cells, 6% neutrophils, 30% lymphocytes, and 2% eosinophils. The centrifugal precipitate smear of the lavage fluid was stained with Wright’s stain and hexamine silver, and *P. jirovecii* cysts were observed under a light microscope (Fig. 2). PJP was diagnosed, and the treatment was changed to sulfamethoxazole and trimethoprim (SMZ-TMP) + clindamycin injection + methylprednisolone injection to treat the PJP. Serum HIV antibody retests were performed using latex agglutination tests and western blotting; however, neither test detected the antibody (Fig. 3). Two weeks after receiving anti-*P. jirovecii* treatment, the patient’s symptoms were mostly relieved, and chest CT revealed that the lung disease was mostly absorbed (Fig. 4). Although the HIV antibody test results were negative or undetermined in three repeated examinations, because the patient was colonised with *P. jirovecii* and he was young and had low CD4^+^ T-cell levels, HIV infection could not be ruled out, and plasma HIV RNA testing was performed. Surprisingly, the results showed that the patient’s viral load was 32,4000 cp/ml, and the retest result also showed a high load of 17,5000 cp/ml; thus, HIV infection was confirmed. After the diagnosis, highly active antiretroviral therapy (HAART) was started with Dolutegravir + Truvada (FTC + TDF). After being hospitalised and observed for 1 week, the patient was discharged. At the time of discharge, the patient had no positive symptoms or signs other than a few scattered rashes on his body.

## Discussion and conclusions

When *P. jirovecii* was discovered, it was found to exist in the tissue in two forms: as a small trophozoite and as a larger cyst form. Therefore, *P. jirovecii* (previously *P. carinii*) was classified as a protozoan for a relatively long time. Based on gene sequence analysis technology, in 2003, Totet et al. found that *P. jirovecii* was a fungus, although its morphology was more similar to that of a protozoan [3]. With developments in technology, in vitro culturing of *P. jirovecii*, which was previously thought to be difficult, was gradually performed. Thus, in 2012, the complete genome sequence of *P. jirovecii* was successfully determined. Examination of the genome showed that it lacked virulence factors, and most amino acid biosynthesis enzymes showed a reduced guanine-cytosine (GC) content and size. This study suggested that *P. jirovecii* mainly infects the lungs and occurs only in immunodeficient patients [4]. Prior to the discovery of AIDS in the 1980s, PJP was considered a rare disease that was only found in patients with severe malnutrition, preterm infants, or patients with leukaemia or other haematological malignancies [5]. After the 1980s and with the continuous increase in AIDS incidence, the number of PJP cases increased rapidly. In many countries, PJP is the most common opportunistic infection among AIDS patients. Studies have shown that in AIDS patients, the *P. jirovecii* colonisation rate can reach 69% [6]. The current evidence, especially from genetic epidemiological studies, has shown that *P. jirovecii* transmission is primarily mediated and spread among people through the air. Individuals colonised with PJP or the *P. jirovecii* population are the infection source for this disease [7–9]. Because PJP is a severe pulmonary infectious disease, its accurate and timely diagnosis is important. PJP is clinically characterised by progressive dyspnoea, coughing, minimal sputum, and fever. It has limited specificity on chest CT and is mainly characterised by diffuse ground glassy lesions in the lungs, which can be uniformly distributed or incorporated with a mosaic sign. As the disease progresses, lobular septum thickening can be superimposed on the ground glass-like lesions, showing a paved stone-like appearance. Because *P. jirovecii* is mainly spread via airway inhalation, the lesions may be more common in the upper lungs [10–12]. Similar chest CT findings are noted for other diseases, such as interstitial pneumonia, cytomegalovirus pneumonitis, lymphoma lung lymph node metastasis, alveolar proteinosis, and allergic or eosinophilic infiltrating alveolitis, but cytomegalovirus pneumonia is the main disease that distinguishes immunodeficient patients. Because many difficulties exist in culturing *P. jirovecii* in vitro, detecting *P. jirovecii* in airway specimens by microscopic examination remains the gold standard for diagnosing PJP. The sensitivity of BALF microscopy is as high as 98% for AIDS patients with PJP [8, 13]. Because PJP is one of the most common opportunistic infections and is common in AIDS patients, it is important to determine whether the patient has HIV infection. Conversely, patients who are clinically diagnosed with PJP should be routinely screened for HIV serum antibodies as well as CD4^+^ and CD8^+^ T-cell counts to determine whether they are infected with HIV as early as possible so that they can receive the earliest possible treatment. Although HIV treatment remains a worldwide problem, early treatment is effective in controlling the disease, and some patients can even achieve a long-term state of asymptomatic survival. In China, the current diagnosis and treatment of AIDS are implemented in accordance with the “Guidelines for AIDS Prevention and Control, Third Edition (2015 edition)”. According these guidelines, HIV1/2 antibody testing is the gold standard for HIV testing and includes preliminary screening and supplementary tests. Preliminary HIV screening tests include ELISA, latex agglutination, colloidal selenium, and gelatine particle agglutination tests, and the supplementary test is a western blotting assay. The test can be repeated using two different principles or reagents from different manufacturers. If all of the results are negative, the patient is determined to be HIV-seronegative [1]. In the case reported here, the ELISA and latex agglutination assay results were negative, and the HIV antibody could not be detected by western blotting, which indicated that this patient was HIV-seronegative. However, when the HIV nucleic acid in the patient’s serum was quantified, the viral load was found to be 32,4000 cp/ml. This was confirmed by retesting; thus, the case was identified as an HIV-seronegative HIV infection. The number of CD4^+^ T-cells in this patient was extremely low, with the presence of a severe opportunistic infection, and the patient was in the AIDS period. For patients within the non-HIV-infection window, HIV-seronegative infection is rare. Twenty-seven cases have been previously reported. In China, Zhang et al. [2] reported one case of HIV-seronegative AIDS in a patient with KS, and the case presented here is the second HIV-seronegative case reported in China. HIV seronegativity in HIV patients occurs mainly during the “window period” and in HIV patients with early HAART. Other than these patients, HIV-seronegative patients are extremely rare. Unfortunately, the reason why these patients are HIV seronegative is unclear. It is partly related to the emergence of rare subtypes, such as HIV groups M and N [14]; however, it is generally believed to be closely related to the host’s immune status. After reviewing the literature, the use of immunosuppressive drugs, such as mycophenolate mofetil, was reported to suppress AIDS humoral immunity and lead to either the absence of or a low titre of HIV antibodies, which cannot be detected [15]. In patients with diseases that result in low humoral immunity levels, such as those complicated with hypogammaglobulinemia, the HIV antibody is undetectable [16]. HIV-specific helper T-cells are T-cells labelled by CD4^+^. CD4^+^ T-cells play a key role in cellular immunity and humoral immune regulation of HIV-infected patients. CD8^+^ T-cells are a group of cytotoxic T lymphocytes (CTLs) that express the CD8 glycoprotein and play an important role in HIV infection after the acute and chronic phases. CD4^+^ T-cells activate CD8^+^ T-cells via dendritic cells. Dendritic cells play a very important role in maintaining the function of memory and effector CD8^+^ T-cells [17–19]. In acute HIV infection, a strong CTL response occurs at the peak of viraemia, which occurs much earlier than production of any neutralising antibody. During this period, a small number of naive CD8^+^ T-cells rapidly differentiate into HIV-specific CD8^+^ T-cells, which can be hydrolysed by HIV-infected cells through identification and secretion of perforin. These cells can produce various cytokines and chemokines to inhibit virus replication and enhance the immune response. They can also initiate the apoptosis of target cells [20–22]. CD4^+^ T-cells are the main target cells of HIV infection and destruction, which can occur in the early stage of infection. In vitro studies have also found that HIV-specific CTLs can hydrolyse CD4^+^ T-cells that are infected by HIV, and these attacks lead to further reduction in the number of CD4^+^ T-cells [19, 23]. In addition to the regulatory function of CD8^+^ T-cells, various subgroups of CD4^+^ T-cells involved in the humoural immunity play a crucial role in facilitating the response of B lymphocytes. Normally functioning and adequate numbers of activated CD4^+^ T-cells can promote formation and maturation of germinal centres, antigen presentation, differentiation, migration, and maturation of B lymphocytes and plasma cells, and help maintain the stability of memory B lymphocytes [24–26].Many studies have found that the number of CD4^+^ T-cells had the greatest effect on HIV antibody titres. Studies have shown that in early disease stages, when the reduction in CD4^+^ T-cell numbers is insignificant, and in patients who are not treated with antiretroviral therapy and exhibit plasma viral loads less than 50 copies/ml, the serum-HIV antibody could maintain a low titre, and as the disease progressed, the HIV antibody titre would gradually increase [27, 28]. As the disease condition worsened, the number of CD4^+^ T-cells could be reduced to very low values. Because B lymphocytes must be activated by CD4^+^ T-cells to signal production of the appropriate antibodies, extremely low CD4^+^ T-cell levels may disrupt this signal transmission, thus severely suppressing humoral immunity and ultimately causing an extremely low serum antibody titre that cannot be detected. An interesting phenomenon is that in some HIV-seronegative patients, after initiating effective antiretroviral therapy and recovering the CD4^+^ T-cell level to a certain extent, the serum HIV antibody could be detected [29]. Compared to HIV-positive patients, HIV-seronegative patients deteriorate faster and have higher mortality [30]. If an HIV-seronegative patient is misdiagnosed, the consequences will undoubtedly be catastrophic. In China, detection of plasma HIV RNA has not been routinely implemented as a preliminary screening test. However, as additional cases are being reported, it is believed that plasma HIV RNA detection will receive increased attention. Regarding the case in this report, it is worth noting that as clinicians, our thinking should not be limited by the examination results. Although the serum HIV antibody was undetected in this patient after multiple screenings and confirmations, the AIDS diagnosis must still be considered. As technology advances, plasma HIV RNA testing can perhaps be routinely performed as an initial test, and HIV-infected patients who are HIV-seronegative may not be as rare as is currently thought.

## Additional files

Additional file 1: Plasma HIV load test reports: the results showed that the patient’s viral load was 32,4000 cp/ml(19 December 2017) , and the retest result also showed a high load of 17,5000 cp/ml(29 December 2017). (PDF 435 kb)

Additional file 2: HIV antibody test report: HIV antibody detected by western blotting(instruments: biological safety cabinet, haier company; automatic western blotting instrument, beetblot 48). Compared with the control group, HIV antibody could not be detected in all three groups. (PDF 309 kb)

## Data Availability

The datasets used during the current article are available from the corresponding author on reasonable request and the materials most important for this article can be available in the section of Additional files 1 and 2.

## Abbreviations

AIDS: Acquired Immune Deficiency Syndrome
BALF: Bronchoalveolar lavage fluid
CT: Computed tomography
CTLs: Cytotoxic T lymphocytes
ELISA: Enzyme-linked immunosorbent
FTC: Emtricitabine
GC: Guanine-cytosine
GCs: Germinal centers
HAART: Highly active antiretroviral therapy
HIV: Human immunodeficiency virus
KS: Kaposi sarcoma
LDH: a serum lactic dehydrogenase
PCO_2_: Pressure of carbon dioxide
PJP: *Pneumocystis jirovecii* pneumonia
PO_2_: Pressure of oxygen () of 62 mmHg
SMZ: Sulfamethoxazole
TDF: Tenofovir
TMP: Trimethoprim

## References

[1] AIDS Professional Group, Society of Infectious Disease, Chinese Medical association (2015). Third edition of the guidelines for diagnosis and treatment of HIV/AIDS(2015). Chin J Clin Infect Dis.

[2] Zhang H, Wang HL, Zhong DR, Liu Y, Li NN, Zhang W, Xiao Y, Li TS (2017). Fatal pulmonary Kaposi sarcoma in an HIV seronegative AIDS patient. Clin Respir J.

[3] Totet A, Latouche S, Duwat H, Magois E, Lacube P, Pautard JC, Schmit JL, Jounieaux V, Roux P, Raccurt C (2003). Multilocus genotyping of Pneumocystis jirovecii in patients developing diverse forms of parasitism: implication for a wide human reservoir for the fungus. J Eukaryot Microbiol.

[4] Cisse OH, Pagni M, Hauser PM (2012). De novo assembly of the Pneumocystis jirovecii genome from a single bronchoalveolar lavage fluid specimen from a patient. MBio.

[5] Kovacs JA, Masur H (2009). Evolving health effects of Pneumocystis: one hundred years of progress in diagnosis and treatment. JAMA.

[6] Huang L, Crothers K, Morris A, Groner G, Fox M, Turner JR, Merrifield C, Eiser S, Zucchi P, Beard CB (2003). Pneumocystis colonization in HIV-infected patients. J Eukaryot Microbiol.

[7] Helweg-Larsen J, Lee CH, Jin S, Hsueh JY, Benfield TL, Hansen J, Lundgren JD, Lundgren B (2001). Clinical correlation of variations in the internal transcribed spacer regions of rRNA genes in Pneumocystis carinii f.sp. hominis. AIDS.

[8] Beck JM, Cushion MT (2009). Pneumocystis workshop: 10th anniversary summary. Eukaryot Cell.

[9] Phipps LM, Chen SC, Kable K, Halliday CL, Firacative C, Meyer W, Wong G, Nankivell BJ (2011). Nosocomial Pneumocystis jirovecii pneumonia: lessons from a cluster in kidney transplant recipients. Transplantation.

[10] Boiselle PM, Crans CJ, Kaplan MA (1999). The changing face of Pneumocystis carinii pneumonia in AIDS patients. AJR Am J Roentgenol.

[11] Fujii T, Nakamura T, Iwamoto A (2007). Pneumocystis pneumonia in patients with HIV infection: clinical manifestations, laboratory findings, and radiological features. J Infect Chemother.

[12] Kuhlman JE, Kavuru M, Fishman EK, Siegelman SS (1990). Pneumocystis carinii pneumonia: spectrum of parenchymal CT findings. Radiology.

[13] Tasaka S (2015). Pneumocystis pneumonia in human immunodeficiency virus-infected adults and adolescents: current concepts and future directions. Clin Med Insights Circ Respir Pulm Med.

[14] Aboulafia DM (2000). The epidemiologic, pathologic, and clinical features of AIDS-associated pulmonary Kaposi's sarcoma. Chest.

[15] Jurriaans S, Sankatsing SU, Prins JM, Schuitemaker H, Lange J, Van Der Kuyl AC, Cornelissen M (2004). HIV-1 seroreversion in an HIV-1-seropositive patient treated during acute infection with highly active antiretroviral therapy and mycophenolate mofetil. AIDS.

[16] Neto R, Guimaraes MC, Moya MJ, Oliveira FR, Louzada PJ, Martinez R (2000). Hypogammaglobulinemia as risk factor for Cryptococcus neoformans infection: report of 2 cases. Rev Soc Bras Med Trop.

[17] Ridge JP, Di Rosa F, Matzinger P (1998). A conditioned dendritic cell can be a temporal bridge between a CD4+ T-helper and a T-killer cell. Nature.

[18] Walter EA, Greenberg PD, Gilbert MJ, Finch RJ, Watanabe KS, Thomas ED, Riddell SR (1995). Reconstitution of cellular immunity against cytomegalovirus in recipients of allogeneic bone marrow by transfer of T-cell clones from the donor. N Engl J Med.

[19] Zajac AJ, Blattman JN, Murali-Krishna K, Sourdive DJ, Suresh M, Altman JD, Ahmed R (1998). Viral immune evasion due to persistence of activated T cells without effector function. J Exp Med.

[20] Meylan PR, Guatelli JC, Munis JR, Richman DD, Kornbluth RS (1993). Mechanisms for the inhibition of HIV replication by interferons-alpha, −beta, and -gamma in primary human macrophages. Virology.

[21] Emilie D, Maillot MC, Nicolas JF, Fior R, Galanaud P (1992). Antagonistic effect of interferon-gamma on tat-induced transactivation of HIV long terminal repeat. J Biol Chem.

[22] Yang OO, Kalams SA, Trocha A, Cao H, Luster A, Johnson RP, Walker BD (1997). Suppression of human immunodeficiency virus type 1 replication by CD8+ cells: evidence for HLA class I-restricted triggering of cytolytic and noncytolytic mechanisms. J Virol.

[23] Yang OO, Kalams SA, Rosenzweig M, Trocha A, Jones N, Koziel M, Walker BD, Johnson RP (1996). Efficient lysis of human immunodeficiency virus type 1-infected cells by cytotoxic T lymphocytes. J Virol.

[24] Fenyo EM, Albert J, McKeating J (1996). The role of the humoral immune response in HIV infection. AIDS.

[25] Miles B, Miller SM, Connick E (2016). CD4 T follicular helper and regulatory cell dynamics and function in HIV infection. Front Immunol.

[26] Vinuesa CG, Linterman MA, Yu D, MacLennan IC (2016). Follicular helper T cells. Annu Rev Immunol.

[27] Bailey JR, Lassen KG, Yang HC, Quinn TC, Ray SC, Blankson JN, Siliciano RF (2006). Neutralizing antibodies do not mediate suppression of human immunodeficiency virus type 1 in elite suppressors or selection of plasma virus variants in patients on highly active antiretroviral therapy. J Virol.

[28] Hatano H, Delwart EL, Norris PJ, Lee TH, Dunn-Williams J, Hunt PW, Hoh R, Stramer SL, Linnen JM, McCune JM (2009). Evidence for persistent low-level viremia in individuals who control human immunodeficiency virus in the absence of antiretroviral therapy. J Virol.

[29] Douek DC, Brenchley JM, Betts MR, Ambrozak DR, Hill BJ, Okamoto Y, Casazza JP, Kuruppu J, Kunstman K, Wolinsky S (2002). HIV preferentially infects HIV-specific CD4+ T cells. Nature.

[30] Spivak AM, Sydnor ER, Blankson JN, Gallant JE (2010). Seronegative HIV-1 infection: a review of the literature. AIDS.

