# Supplementary material for: A rare case of an HIV-seronegative AIDS patient with Pneumocystis jirovecii pneumonia
Source: BMC Infect Dis. 2019 Jun 14;19:525. doi: 10.1186/s12879-019-4143-8 (PMC6570926; doi:10.1186/s12879-019-4143-8)
Supplement: Supplementary file 1 — Plasma HIV load test reports: the results showed that the patient’s viral load was 32,4000 cp/ml(19 December 2017) , and the retest result also showed a high load of 17,5000 cp/ml(29 December 2017). (PDF 435 kb) [file 12879_2019_4143_MOESM1_ESM.pdf]

Plasma HIV load test reports: the results showed that the patient's viral load was 32,4000 cp/ml, and the retest result also showed a high load of 17,5000 cp/ml.

台州市疾病预防控制中心[illegible]

共 / [illegible]第 / 页

样品名称 血浆　　样品数量 1 份　　实验编号 VL171961

收样日期 2017-12-19　　检测日期 2017-12-19　　报告日期 2017-12-19

送检单位 台州恩泽医疗中心恩泽医院

检测项目 HIV-1 病毒载量

姓　　名 [redacted]　性别 男　　年龄 22 岁　　身份证号 [redacted]

电　　话 [redacted]

检测结果

序号	实验编号	姓名	样品编号	治疗编号	HIV-1 病毒载量(cp/ml)	采血日期	备注
1	VL171961	[redacted]	17TZV008	/	324000	2017-12-18	

备注：TND 为目标未被检测到；failed，invalid 为样本量少于 1mL 或样本不合格（有凝块或其他）造成检测失败。

本检测结果只对该送检标本负责。

检测人：李桂霞

日期：2017年 12月 19日

复核人：[illegible]

日期：2017年 12月 19 日

台州市疾病预防控制中心检测单

共 1 页 第 1 页

样品名称 血浆 样品数量 1份 实验编号 VL172077

收样日期 2017-12-29 检测日期 2017-12-29 报告日期 2017-12-29

送检单位 台州恩泽医疗中心恩泽医院

检测项目 HIV-1 病毒载量

姓 名 [redacted] 性别 男 年龄 22岁 身份证号 [redacted]

电 话 [redacted]

检测结果

序号	实验编号	姓名	样品编号	治疗编号	HIV-1 病毒载量(cp/ml)	采血日期	备注
1	VL172077	[redacted]	17TZV010	/	175000	2017-12-28	

备注：TND 为目标未被检测到；failed，invalid 为样本量少于 1mL 或样本不合格（有凝块或其他）造成检测失败。

本检测结果只对该送检标本负责。

检测人：李桂霞

日期：2017 年 12 月 29 日

复核人：沈伟伟

日期：2017 年 12 月 29 日
