# Supplementary material for: A rare case of an HIV-seronegative AIDS patient with Pneumocystis jirovecii pneumonia
Source: BMC Infect Dis. 2019 Jun 14;19:525. doi: 10.1186/s12879-019-4143-8 (PMC6570926; doi:10.1186/s12879-019-4143-8)
Supplement: Supplementary file 2 — HIV antibody test report: HIV antibody detected by western blotting(instruments: biological safety cabinet, haier company; automatic western blotting instrument, beetblot 48). Compared with the control group, HIV antibody could not be detected in all three groups. (PDF 309 kb) [file 12879_2019_4143_MOESM2_ESM.pdf]

Raw record of HIV antibody detecting by western blotting.

台州恩泽医疗中心(集团)

恩泽医院检验科艾滋病确证实验室

HIV 抗体检测 原始记录

文件编号：EZYY-JY-AZ-JL-05

版本号：A/0 第 1 页 共 1 页

样品编号 EZ2017049-EZ2017051 收样日期 2017.12.18

检测项目 HIV 抗体 检测日期 2017.12.18

检测地点 艾滋病确证实验室 检测环境（温、湿度） 20 ℃ 38%RH

检测依据（方法）全国艾滋病检测技术规范（2015 修订版）

检测仪器名称、型号及编号☐生物安全柜海尔 HR40-ⅡA2 EZYY-JY-SB-SM-A-60 ☐全自动蛋白印迹仪 BeetBlot 48 EZYY-JY-SB-SM-A-57

仪器状况 正常☑ 异常☐

检验记录与结果

免疫印迹法（WB）

1 检测方法：免疫印迹法（WB），（具体操作按试剂使用说明书进行）

2 试剂来源：☑上海英旻泰生物技术有限公司

人类免疫缺陷病毒（HIV1+2 型）抗体检测试剂盒（免疫印迹法）

IMT HIV-1/2 Blot 确证试剂盒

批号：IMT1613001 有效期：2019-01-11 。

结果及报告：

实验编号	姓名	实验结果				结果报告 HIV-1 抗体
		快速法	ELISA 法	WB 法		
				条带号	WB 法带型	
EZ2017049	[redacted]	/	/	31	p24,p17	不确定
EZ2017050	[redacted]	/	/	32	p24,p17	不确定
EZ2017051	[redacted]	/	/	33	p24,p17	不确定
		/	/			
		/	/			
强阳性对照				34	gp160,gp120,p66,p51/55,gp41,p31,p24,p17,HIV-2 型提示带	阳性
弱阳性对照				35	gp160,p66,p51/55,p31,p24,HIV-2 型提示带	阳性
阴性对照				36	无 HIV 抗体特异性条带	HIV 抗体阴性
